# Analysis of the mutations in exon 10 of *MEFV * gene in patients with premature coronary heart disease in west Azerbaijan province of Iran

**DOI:** 10.15171/jcvtr.2018.03

**Published:** 2018-03-17

**Authors:** Morteza Bagheri, Kamal Khadem-Vatani, Mir Hossein Seyed Mohammad Zad, Isa Abdi Rad, Behzad Rahimi, Alireza Rostamzadeh, Mojtaba Godarzi, Shabnam Ashena

**Affiliations:** ^1^Cellular and Molecular Research Center, Urmia University of Medical Sciences, Urmia, Iran; ^2^Seyyed-al Shohada University Hospital, Urmia University of Medical Sciences, Urmia, Iran

**Keywords:** MEFV, PCHD, Mutations

## Abstract

***Introduction:*** Premature coronary heart disease (PCHD) affects public health and leads to death. PCHD has several genetic and environmental risk factors. The aim of this study was to analysis of the mutations in exon 10 of *MEFV * gene in patients with PCHD in West Azerbaijan province of Iran.

***Methods:*** Totally 41 PCHD patients who were admitted to the cardiology unit of Sayedoshohada hospital (Urmia, Iran) enrolled in the study. Selection of the patients was done based on the strict criteria, that is, who had a minimum of one angiographically documented coronary artery with the stenosis of 50%. Mutations in exon 10 of *MEFV * gene were found by direct sequencing.

***Results:*** V726A, M680I, K695R, and A744S mutations with 2.44%, 1.22%, 1.22%, and 1.22%, allelic frequency were found, respectively. Five patients (12.2%) with PCHD carried at least one mutated MEFV allele. Heterozygote V726A was the most frequent mutation among tested cases (4.88%), followed by heterozygote M680I, heterozygote K695R, and heterozygote A744S.

***Conclusion:*** The results of the present study imply that the frequency of the *MEFV * gene exon 10 is significantly high in PCHD patients. This is the first report in its own kind in clinically diagnosed PCHD pa­tients of Iranian Azeri Turkish population.

## Introduction

Premature Coronary Heart Disease (PCHD), also defined as premature coronary artery disease (PCAD) affects public health and leads to death.^1^ CAD has several genetic and environmental risk factors. The prevalence of CAD became increased in developing countries such as Iran.^1^ CAD is responsible for approximately 50 percent of all deaths per year in Iran.^1^ CAD as a multifactorial disease is influenced by gender. CAD prevalence is about 6.9% and 6% in men and women, respectively.^2^ Several traditional risk factors influences the CAD related disability and mortality such as obesity, end stage renal disease, diabetes mellitus, smoking, dyslipidemia, physical inactivity, metabolic syndrome, family history of PCAD and systemic anatomic vascular disorders.^3,4^ The pathogenesis of CAD is poorly understood.^3^ CAD is a common form of coronary atherosclerosis. CAD is the result of creation of atherosclerotic plaques within coronary vessels that leads to a heart attack or unexpected cardiac-fatality.^4^ Reduction of coronary artery flow is under the impact of impediment severity and the rapidity of its expansion.^5^ It may occur at any time of life, but most commonly happens in young asymptomatic individuals.^6^ Management of CAD is dependent on the identification of genetic and environmental factors such as gene-gene and gene-environment interactions.^7^ There is an interest to find new biomarkers that can judge CAD risk and therapeutic efficiency. Large bodies of biomarkers have been evaluated in CAD such as circulating and inflammation-associated microRNAs^8^, Urinary proteomic biomarkers,^9^ and inflammatory biomarkers.^10^ Familial Mediterranean fever (FMF) as a multisystem disease is an autosomal recessive disease that predominantly influences various tissues of the body such as gastrointestinal tract, heart, testis, liver, spleen, lungs, and kidneys. Clinical presentation of FMF includes abdominal pain, muscle pain, rash and fever, amyloidosis, vasculitis, and infertility.^11^ The *MEFV* gene is mapped on the short arm of chromosome 16 (16p13.3) and has 10 exons and encodes pyrin (marenostrin) with 781 amino acid.^12^ The role of pyrin was not well understood, but it is supposed that to act as anti-inflammatory mediator.^13^ Basar et al revealed for the first time that MEFV mutations could be as a risk factor for early CHD.^14^ More than 180 mutations have been recognized in the *MEFV* gene,^15^ but CHD-associated MEFV gene mutations were not studied in Iranian population. Mutations in exon 10 of *MEFV* gene has clinical significance in our population.^16^ The aim of this study was to analyze the mutations in exon 10 of *MEFV* gene in patients with PCHD in West Azerbaijan province of Iran.

## Materials and Methods

Totally 41 CAD patients who were admitted in the cardiology unit of Sayedoshohada hospital (Urmia, Iran) enrolled in the study. This study was done at Urmia University of Medical Sciences (Urmia, Iran). PCHD was identified with an age of onset of CHD ≤55 years in males and ≤65 years in females.^17^ Selection of the patients was done based on strict criteria, that is, who had a minimum of one angiographically documented coronary artery with the stenosis of 50%.^18^ Diagnosis of CAD was confirmed by electrocardiography, coronary angiography, and echocardiography.^18^ None of our cases was clinically diagnosed with FMF. Exclusion criteria were high blood pressure, diabetes, and smoking. Patients were evaluated by an expert cardiologists based on the accepted criteria. Each patient was informed about the contents and aims of the study. 2-3 ml blood sample were obtained in EDTA-containing tubes for extraction of DNA. Blood samples were preserved in -20^°^C till DNA extraction. DNA extraction was carried out using standard “salting out” method,^19^ and then was preserved in -80^°^C till PCR. In our samples, the purity of DNA extracts was confirmed by measuring absorption at 260 nm and 280 nm in a Biophotometer (Ependorf AG, Germany). Mutations in exon 10 were found by direct sequencing of PCR products using two sets of primers including MEFV F: 5´-ccc atg gac ccc tac cta gg- 3´and MEFV R: 5´-aag aga gat gca gtg ttg ggc-3´.^14^ The PCR program was as: 94 °C for 4 minutes; 30 cycles: 94°C for 1 minute and 58°C for 30 seconds. PCR reactions were carried out in 25 μL solution: 100 ng of DNA, 1x reaction buffer 5 pmol of each primer, 200 μmol of each dNTPs, 0.2 unit of Taq DNA polymerase, and 1.5 mmol MgCl_2_. PCR products were analyzed via electrophoresis on 2% agarose gel stained with CinnaGen DNA safe Stain (CinnaGen Co. Tehran, Iran). Presence of a 617(bp) fragment was monitored by UV transilluminator. Subsequently direct sequencing of the PCR products was carried out in an ABI 730XL DNA analyzer (Applied Biosystems). Chromas Lite version 2.1.1 (2012) was used for chromatogram visualization of sequenced DNA fragments (Chromas Lite version 2.1 (2012), Technelysium Pty Ltd, South Brisbane, Queensland, Australia). Descriptive statistics were used to report the frequency of the mutations in this study.

## Results

**Figure 1:**
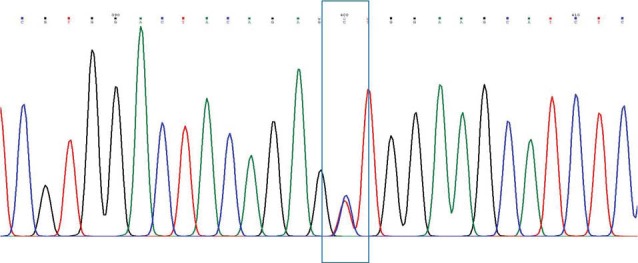
Schematic representation of the V726A mutation in exon 10 of *MEFV* gene. Red color graph represent the T allele and blue color graph represent the C allele in suspected DNA locus (c.2177T>C).

**Figure 2:**
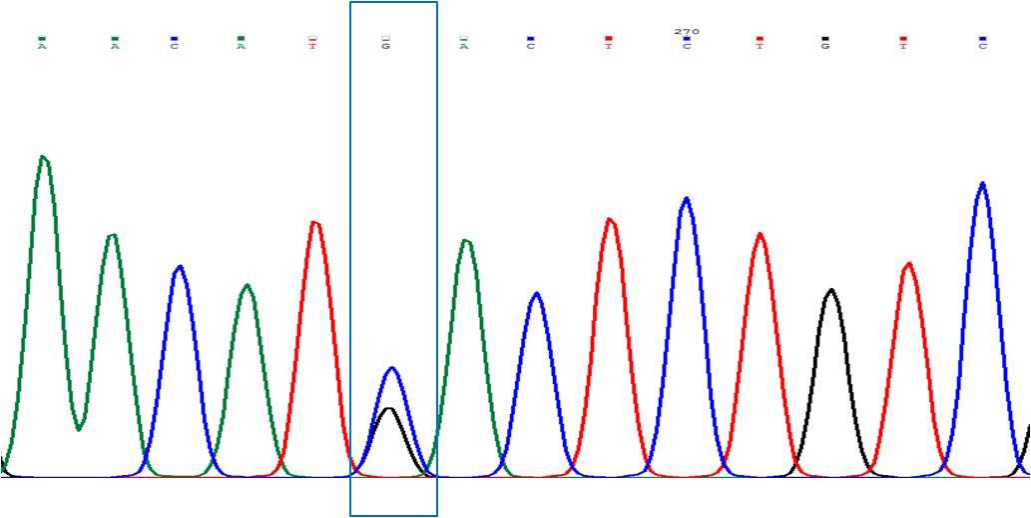
Schematic representation of the M680I mutation in exon 10 of *MEFV* gene. Black color graph represent the G allele and blue color graph represent the C allele in suspected DNA locus (c.2040G>C).

**Figure 3:**
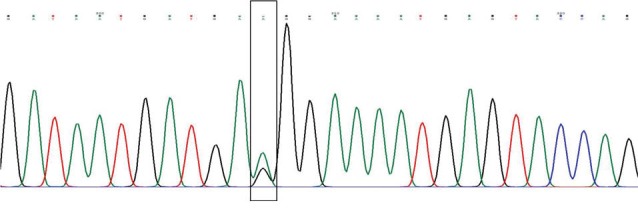
Schematic representation of the K695R mutation in exon 10 of *MEFV* gene. Green color graph represent the A allele and black color graph represent the G allele in suspected DNA locus (c.2084A>G).

**Figure 4:**
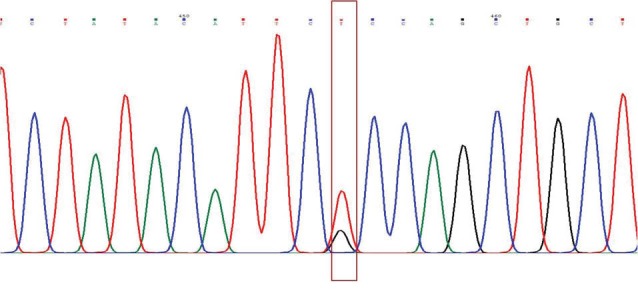
Schematic representation of the A744S mutation in exon 10 of *MEFV* gene. Black color graph represent the G allele and red color graph represent the T allele in suspected DNA locus (c.2230G>T).

The findings of this study are shown in Figures 1-4. Five patients (12.2%) with PCHD carried at least one mutated MEFV allele. Heterozygote V726A was the most frequent mutation among tested cases (4.88%), followed by heterozygote M680I, heterozygote K695R, and heterozygote A744S. The frequencies of cases and chromosomes with at least one mutation are reported in Table 1. The average age of our patients was 45.25±5.28.

**Table 1:** Studied mutations and the frequencies of cases and chromosomes with at least one mutation in this study

**Protein Mutation**	**Nucleotide Mutation**	**Amino Acid Change**	**Genotype**	**Cases (%)**	**Chromosomes (%)**
V726A	c.2177T>C	V[Val]/A[Ala]	Heterozygote	2(4.88)	2(2.44)
M680I	c.2040G>C	M[Met]/I[Ile]	Heterozygote	1(2.44)	1(1.22)
K695R	c.2084A>G	K[Lys]/R[Arg]	Heterozygote	1(2.44)	1(1.22)
A744S	c.2230G>T	A[Ala]/S[Ser]	Heterozygote	1(2.44)	1(1.22)

## Discussion

CAD is a leading killer but preventable by reduction of risk factors, so, the risk factors of CAD should be determined.^20^ The total risk of CAD is dependent on the interaction of genetic and environmental risk factors. Most of these risk factors appear to promote the CAD through atherosclerosis.^21^ Atherosclerosis is the result of chronic inflammation and is accelerated by risk factors such as hypertension, hyperlipidemia particularly low-density lipoproteins (LDL), type 2 diabetes mellitus, obesity, cigarette smoking, and genetic susceptibility^21^. Some risk factors are under genetic control and a positive family history is identified as an important predictor of CAD.^22^ The genetic factors are involved in 40%–50% of CAD cases, and several studies showed many genetic loci that predispose the patients to CAD.^22^ Also, large bodies of single-nucleotide polymorphisms (SNPs) have been tested in human diseases.^13,23-27^ In coronary atherosclerosis, pathophysiological changes are slow and lead to intimal thickening and formation of atherosclerotic plaques.^28^ In coronary vessels, the immune response contains increased expression of adhesion molecules and inflammatory cytokines.^29^ These processes lead to the development of plaques and unexpected progression of plaques and variable clinical findings.^30^ Numerous genetic factors influence the CAD susceptibility with different effect size, and determination of genetic biomarkers such as CAD-associated RNA-based biomarkers can provide novel methods for the prediction and management of CAD.^31^ In this regard, role of several cytokines (such as IL-17, IL-18) are identified in endothelial dysfunction, oxidative stress and over-production of adhesion molecules.^32,33^ The focus of this study was to evaluate the role of *MEFV* gene mutations on the exon 10 in the PCHD patients. In this study, V726A, M680I, K695R, and A744S mutations were found. The results of the present study were in agreement with Basar et al, and imply that the frequency of MEFV mutations have significantly increased in the PCHD patients. Bonyadi et al determined the *MEFV* gene mutations among Iranian Azeri Turkish general population (the same ethnic group as of our study) that indicated none of tested individuals were carriers for M694V, M694I, and M680I, whereas 1.75% of the chromosomes carried the V726A mutations. K695R and A744S mutations are less common.^34^ In our study, 12.2% of cases had a mutation in exon 10 of *MEFV* gene, which is in agreement with Basar et al. M680I, K695R, and A744S mutations, which were not found or found with very low frequency in the general population, account for 3.66% of chromosomes with at least one mutation. Present study has some limitations including the small number of tested patients, poor quality of registry data regarding family medical history, other contributing risk factors such as psychosocial factors, stress and physical activity. Future study, with large number of samples, is essential to confirm these findings.

## Conclusion

The present study is the first report in its own kind and implies that the frequencies of MEFV mutations on the exon 10 are increased significantly in PCHD patients.

## Competing interests

The authors declare that they have no competing interests.

## Ethical approval

Urmia University of Medical Sciences Research Ethics Committee has approved all stage of this study (Ir.umsu.rec.1394.138).
